# Adaptive Autophagy Offers Cardiorenal Protection in Rats with Acute Myocardial Infarction

**DOI:** 10.1155/2020/7158975

**Published:** 2020-06-20

**Authors:** Zhendong Feng, Han Xue Jiang, Huiyang Chen, Yu Ning Liu, Yahui Wang, Rui Bing Yang, Xueting Han, Chen Hui Xia, Ze Bing Zhu, Hongcai Shang, Aiming Wu, Wei Jing Liu

**Affiliations:** ^1^Key Laboratory of Chinese Internal Medicine of Ministry of Education and Beijing, Dongzhimen Hospital Affiliated to Beijing University of Chinese Medicine, Beijing 100700, China; ^2^Beijing Chinese Medicine Hospital Pinggu Hospital, Beijing 101200, China; ^3^Renal Research Institution, Beijing University of Chinese Medicine, Beijing 100700, China; ^4^Institute of Nephrology, Zhanjiang Key Laboratory of Prevention and Management of Chronic Kidney Disease, Guangdong Medical University, Zhanjiang, Guangdong 524001, China

## Abstract

**Objective:**

Understanding the multifactorial changes involved in the kidney and heart after acute myocardial infarction (AMI) is prerequisite for further mechanisms and early intervention, especially autophagy changes. Here, we discussed the role of adaptive autophagy in the heart and kidney of rats with AMI.

**Methods:**

A rat model of AMI was established by ligating the left anterior descending branch of the coronary artery. Animals were sacrificed at 2 and 4 weeks after the operation to assess the morphological and functional changes of the heart and kidney, as well as the autophagy pathway. In vitro, HK-2 and AC16 cell injuries and the autophagy pathway were assayed after autophagy was inhibited by 3-methyladenine (3-MA) in a hypoxia incubator.

**Results:**

We found that the left ventricular systolic pressure (LVSP) significantly decreased in the model group at weeks 2 and 4. At weeks 2 and 4, the level of urinary kidney injury molecule 1 (uKIM1) of the model group was significantly higher than the sham group. At week 4, urinary neutrophil gelatinase-associated lipocalcin (uNGAL) and urinary albumin also significantly increased. At week 2, microtubule-associated protein 1 light chain 3-II (LC3-II), ATG5, and Beclin1 were significantly elevated in the heart and kidney compared with the sham-operated rats, but there was no change in p62 levels. At week 4, LC3-II did not significantly increase and p62 levels significantly increased. In addition, 3-MA markedly increased KIM1, NGAL, and the activity of caspase-3 in the hypoxic HK-2 and AC16 cell.

**Conclusion:**

Autophagy will undergo adaptive changes and play a protective role in the heart and kidney of rats after AMI.

## 1. Introduction

At present, AMI accounts for the highest proportion of coronary heart disease in China [1]. Studies have shown that kidney injury is considered to be a common detrimental event of AMI and frequently associated with high mortality [2, 3]. What is more, it has been reported that chronic kidney disease occurred in 45–63% of patients with congestive heart failure (CHF), and deterioration of renal function is also an important cause of heart failure, resulting in a poor prognosis, even death [4, 5]. Although cardiorenal syndrome (CRS) has garnered attention since it was defined and classified in 2008, the mechanisms between the heart and kidney are still ambiguous, and better therapeutic intervention to improve outcome is still lacking [6]. Therefore, understanding the multifactorial changes involved in the kidney and heart after AMI is crucially required to explore potential targets for further mechanisms and early intervention.

Autophagy is an effective mechanism for the degradation of harmful intracellular substances such as damaged organelles and misfolded proteins, which maintains cellular homeostasis. It plays a crucial role in the pathogenesis of the kidney and heart diseases [7]. Loss of autophagy-related 5 (ATG5), a key protein in autophagy, causes heart failure in diabetic cardiomyopathy, whereas the enhancement of autophagy can delay the progress of hypoxic heart disease and improve ventricular remodeling [7–9]. In addition, autophagy is induced in acute kidney injury to protect renal tubular epithelial cells from injury [10]. Zhang et al. used rapamycin as an autophagy agonist to treat heart failure rats caused by proximal coronary ligation [11], and they found that autophagy could improve cardiac function and apoptosis. However, how autophagy changes was not detected. Few studies have observed autophagy changes in the heart and kidney at the same time and whether their changes are consistent. More importantly, this is the basis of the use of autophagy in treating cardiorenal syndrome. Considering these facts, in our study, we tested the role of autophagy in the heart and kidney of rats after acute myocardial infarction (AMI). First, we clarify whether the changes are consistent in the heart and kidney, and then elucidate the protective effect of adaptive autophagy.

## 2. Material and Methods

### 2.1. Myocardial Ischemia Models

All experiments and applications in animals were approved by the Standing Committee on Animals at the Dongzhimen Hospital Affiliated to Beijing University of Traditional Chinese Medicine (Protocol Number: 17-12, Beijing, China). Male Sprague-Dawley rats (weight, 200 ± 20 g) in this study were obtained from the Beijing Vital River Laboratory Animal Technology Co. Ltd.. (License No. 11400700368087; Beijing, China). During the experiment, all animals were raised in the animal barrier system of the Key Laboratory of Chinese Internal Medicine of the Ministry of Education, Beijing, with free access to pure water and common feed. Rats with AMI were established by permanent left coronary artery ligation as previously described [12]. The sham-operated group was only given thoracotomy and opened the pericardium without ligation of left anterior descending coronary artery. After the abovementioned operations were completed, the thorax was completely and tightly closed. The electrocardiogram (ECG) and ventilator were disconnected at the end of surgery. All animals were carefully monitored until they regained enough consciousness to allow spontaneous breathing. Compared with preoperative ECG, the ST-segment elevated immediately after ligation, and the pathological Q-waves appeared on the second day after surgery, which indicated establishment of the successful model. These rats that survived after myocardial infarction belonged to the model group. Finally, 0.24 g penicillin (F6062105, Hebei, China) was injected intraperitoneally for three days to prevent infection. Animals were sacrificed at 2 and 4 weeks after the operation.

### 2.2. Detection of Heart and Renal Function

Intubation of the internal left ventricle was conducted to detect cardiac hemodynamics in rats (BL-420S, Chengdu, China) at weeks 2 and 4 [13]. After anesthetizing the rats, with skin preparation and disinfection of the neck, the animals were positioned in the supine position. The anterior cervical muscles were carefully and bluntly separated to expose the right carotid artery. After ligating the proximal and distal ends of the artery, a “T-notch” was cut on the blood vessel. Then, one heparin-f PE catheter (inner diameter, 0.05 mm) was inserted into the right carotid artery and carefully pushed to the left ventricle. The other end of the catheter was connected to the biological function acquisition system (BL-420S; Chengdu, China). After the signal was stabilized for 5 min, the following four indicators were recorded: the left ventricular systolic pressure (LVSP), left ventricular end-diastolic pressure (LVEDP), and maximum positive and negative values of dp/dt (±dp/dt max). At weeks 2 and 4, urine and blood samples were collected. The serum creatinine (SCr), blood urea nitrogen (BUN), uKIM1, uNGAL, and urinary albumin levels were measured according to the manufacturer's instructions (C011-2, C013-2, Nanjing Jiancheng Technology, Nanjing, China; ab119597, ab119602, ab108789, Abcam). Then, renal cortex specimens and the myocardium were processed for morphological studies or for the western blot analysis.

### 2.3. Histological and Immunohistochemical Studies

Kidney and heart sections (3 *μ*m thick) were stained with periodic acid-Schiff (PAS), Masson's trichrome staining, or hematoxylin and eosin (H&E) as previously described [14]. Mouse anti-LC3B (sc-271625 for tissue), mouse anti-p62 (ab56416, Abcam), and horseradish peroxidase-(HRP-)labeled anti-mouse IgG antibodies (PV-9001; Zhongshan Golden Bridge Biotechnology Technology, Beijing, China) were used for tissue immunostaining assays as previously described [15]. Images (15 pictures of each rat) were taken under the A1 HAL 100 microscope (ZEISS Microscopy, Jena, Germany), and each image was taken under the same condition. Expression levels of LC3-II and p62 in each picture were graded on a scale of 1–5 according to the number of dots, and the average of the scores was subsequently calculated. Apoptosis was assessed using a TUNEL apoptosis assay kit (G3250, Promega, Fitchburg, WI, USA) according to the manufacturer's instructions.

### 2.4. Western Blot Analysis

The western blot analysis was performed as previously described [14]. 20 *μ*g of total protein was loaded in a stacking polyacrylamide gel for all of the experiment. In vitro, when the sample was loaded in the gel for testing LC3-II, Beclin1, and *β*-actin, the same sample was loaded in another gel for testing p62 as a parallel experiment. Primary antibodies against LC3B (ab51520, Abcam), p62 (ab56416, Abcam), ATG5 (ab108372, Abcam), Beclin1 (ab207612, Abcam), GAPDH (60004-I-IG, Proteintech), *β* actin (sc-47778, Santa Cruz), and HRP-conjugated secondary antibodies (SA00001-1 and SA00001-2, Proteintech) were used.

### 2.5. Cell Culture and Treatments

AC16 cells were obtained from American Type Culture Collection (ATCC) and cultured in Dulbecco's Modified Eagle's Medium with 15% fetal bovine serum (FBS, HyClone, UT, USA), 100 U/mL penicillin, and 100 mg/ml streptomycin (Life Technologies, Carlsbad, CA, USA). Cell lines were cultured at 37°C in a humidified atmosphere with 5% CO_2_. Human proximal tubular (HK-2) cells (ATCC) were maintained in low glucose DMEM (Gibco, Grand Island, NY, USA) supplemented with 10% FBS (Gibco) under standard conditions. Cells were stimulated with hypoxia and 3-methyladenine (3-MA) (5 mM; Sigma, St. Louis, MO, USA).

### 2.6. Cell Hypoxia Model Establishment

We cultured the cells with low glucose medium in an oxygen chamber with 1% oxygen and 5% carbon dioxide and 94% nitrogen gas for 4 and 24 h. Then, the supernatant liquid of AC16 and HK-2 cells was collected, and the activity of caspase-3 and levels of KIM1 and NGAL were measured by ELISA kits (Abcam).

### 2.7. Statistical Analysis

All statistical tests were performed with SPSS 21.0. All data are expressed as the mean ± standard error of the mean (SEM). Two-group comparisons were conducted using the independent samples *t*-test. The multiple group comparison was performed using ANOVA, followed by the Tukey test. The *p* value was considered as statistically significant if it is less than 0.05.

## 3. Results

### 3.1. Cardiac Function Changes in Rats with AMI

At week 2, compared with the sham group, the left ventricular anterior wall and the apex of the model group had obvious infarction, the infarct area became white, and the apex collapsed. When it came to week 4, the ventricular wall of the model group became thinner, collapsed, and showed extensive pale fibrosis after permanent ligation of the left anterior descending branch. Although we collected gross heart images and heart size, we did not find differences in the heart size between the groups in this study (supplementary data 1A), which may be partly due to left ventricular remodeling. As shown in Table 1, the LVSP significantly decreased in the model group at weeks 2 and 4. There was no change in LVEDP between the sham group and the model group at week 2, while the LVEDP significantly increased in the model group at week 4 and was markedly higher than week 2. The + dP/dt max significantly decreased in the model group at weeks 2 and 4. Compared with the sham group, the -dP/dt max was also significantly attenuated in the model group at weeks 2 and 4.

### 3.2. Renal Function Changes in Rats with AMI

**Figure 1:**
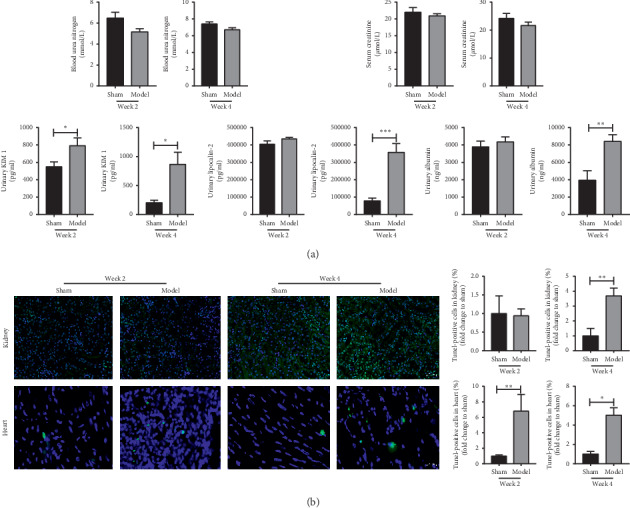
Changes of renal function and apoptosis in rats with heart failure after myocardial infarction (MI). (a) Level of BUN, SCR, KIM-1, NGAL, and urinary albumin (*n* = 7). (b) Apoptosis of the kidney and heart. Each bar represents the mean ± SEM (*n* = 5). ^*∗*^*p* < 0.05, ^*∗∗*^*p* < 0.01, and ^*∗∗∗*^*p* < 0.001.

We found that the renal tubular injury marker uKIM1 in the model group was significantly higher than the sham group at week 2, but there was no increase in the level of uNGAL. While, at week 4, uKIM1, uNGAL, and urinary albumin levels markedly increased in the model group (Figure 1(a)). We did not observe an increase of SCr and BUN levels in the model group.

### 3.3. Histological Changes of the Heart and Kidney in Rats with AMI

Masson's staining showed that the area of fibrosis was greater in the model group at weeks 2 and 4 in the heart, but there was no significant fibrosis in the kidney (supplementary data 1B). These data suggested that fibrosis may not be a major contributor to the kidney damage in this model. In the kidney, tubule dilation, tubule basement membrane separation, and vacuole degeneration were found in the model group by PAS staining. In H&E staining, we observed a lot of inflammatory cells infiltration in the myocardial cells in the model group at week 2 (supplementary data 1B). Apoptosis plays a key role in heart failure and acute kidney injury [10, 16]; so we used TUNEL to evaluate the apoptotic condition. Our TUNEL results indicated that apoptotic myocardial cells were significantly increased in the model group at weeks 2 and 4, and the rate of apoptosis in renal tubular cells also significantly increased at week 4, but did not increase at week 2 (Figure 1(b)).

### 3.4. Autophagy Changes in the Heart and Kidney of Rats with AMI

**Figure 2:**
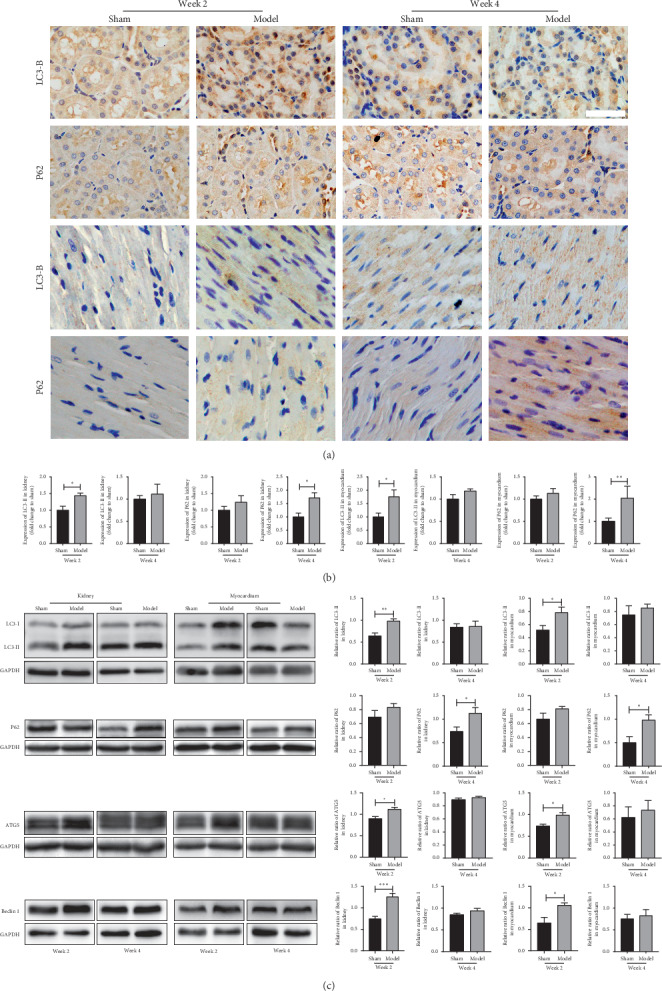
Autophagy changes in the kidney of rats at weeks 2 and 4 after myocardial infarction (MI). (a) Immunohistochemical staining of LC3 and p62 in renal tubular and heart of sham-operated or model rats. (b) Quantitation of LC3-II and p62 in the kidney and heart. (c) Western blot analysis of LC3-II, p62, ATG5, and Beclin1 levels in the kidney and heart. Densitometry was performed for quantification, and the ratio of LC3-II, p62, ATG5, and Beclin1 to GAPDH is expressed as ^*∗*^*p* < 0.05 (IHC: *n* = 6; western blotting: *n* = 5).

Autophagy plays an important role in protecting cardiac function from myocardial damage [8]. Therefore, we detected the changes of autophagy in the kidney and heart at weeks 2 and 4 after AMI. We detected the level of LC3-II and p62 in proximal renal tubules and myocardium by immunohistochemistry and western blotting. At week 2, LC3-II was significantly elevated in the heart and kidney compared with sham-operated rats, while there was no significant increase in p62, which linked the autophagy pathway with ubiquitinated aggregates and was degraded upon autophagosome processing [17]. However, at week 4, we found p62 markedly upregulated both in the heart and kidney, while LC3-II had no significant increase (Figure 2). These results suggested that in the early stage of heart failure after AMI, the induction of autophagy might promote the degradation of p62 [18]. Subsequently, we detected the expression of ATG5 and Beclin1, which were upstream proteins of autophagy. We found that at week 2, the ATG5 and Beclin1 were significantly increased in the model group, while at week 4, the increases were not significant compared with the sham group. Changes in myocardium were consistent with the kidney.

### 3.5. Effects of Autophagy Inhibition on HK-2 and AC16 Cells under Hypoxia Condition

**Figure 3:**
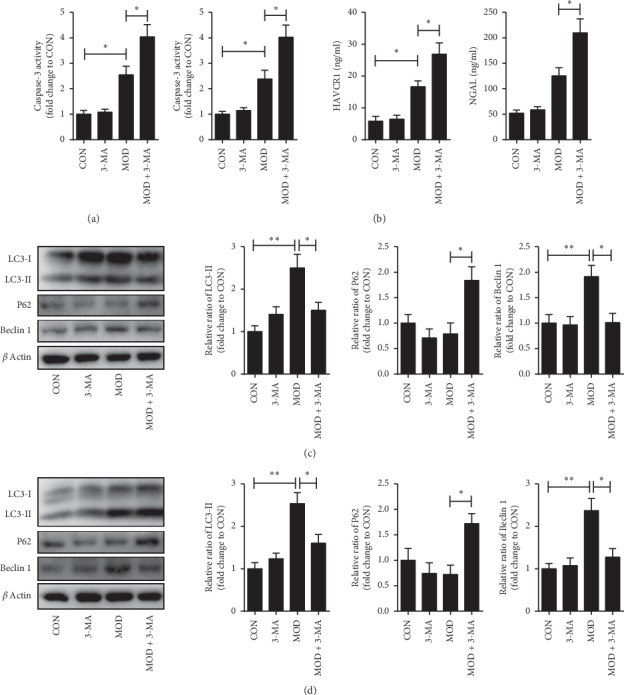
Autophagy inhibition aggravates apoptosis and injury and autophagy changes in AC16 and HK-2 cells. (a) Activity of caspase-3 in AC16 cells. (b) Activity of caspase-3 and Levels of KIM1 and NGAL in HK-2 cells. (c) Western blot analysis of LC3-II, p62, and Beclin1 levels in AC16 cells. (d) Western blot analysis of LC3-II, p62, and Beclin1 levels in the HK-2 cells. CON = normal group. 3-MA = normal + 3-methyladenine. MOD = hypoxic cell group. MOD + 3-MA = hypoxic cell group + 3-methyladenine. ^*∗*^*p* < 0.05 (*n* = 3).

To understand the role of autophagy in the kidney and heart after AMI, we established a model of ischemia and hypoxia in vitro to demonstrate cellular damage and the role of autophagy. We found that the activities of caspase-3 in AC16 and HK-2 cells were significantly increased in the hypoxia group. The levels of KIM1 and NGAL were significantly increased in HK-2 cells (Figures 3(a) and 3(b)). The level of Beclin1 and LC3-II in AC16 and HK-2 cells after 4 hours of hypoxia induction increased dramatically, while the level of p62 did not change significantly. We also observed that there was no significant difference in the above indicators at 24 hours of hypoxia induction (data not listed). After incubated with 3-MA to inhibit autophagy, the level of Beclin1 and LC3-II was significantly declined, while the level of p62 was increased dramatically, both in AC16 (Figure 3(c)) and HK-2 (Figure 3(d)).

## 4. Discussion

In this study, autophagy changes were detected in rats at weeks 2 and 4 after AMI and further discussed the effect of autophagy in vitro. We found that the autophagy in the heart and kidney showed a dynamic process. At week 2 after myocardial infarction, autophagy is adaptively increased. With the time increasing, the levels of autophagy tended to decrease gradually, while the damage gradually worsened, especially the renal function. Much more importantly, in vitro, the injury and apoptosis increased significantly after inhibiting autophagy. The results suggested that the adaptive autophagy protected the kidney and heart from injury after AMI. Moreover, this study was instructive for autophagy intervention in the early clinical stage of AMI.

First, we established the AMI model by ligating the left anterior descending branch of the coronary artery, which is similar to the pathogenesis of human cardiac insufficiency and reproducible. Studies have indicated that the pathogenic mechanisms of interaction between the heart and kidney may correlate with hemodynamic perturbation, nonhemodynamic pathways, the sympathetic nervous system, the renin-angiotensin-aldosterone system, inflammation, and oxidative stress [19–22]. In the acute stage of myocardial infarction, inflammation is the main cause of heart injury. The acute phase is usually considered as one week within MI. After two weeks of myocardial infarction, the ischemic myocardium gradually became fibrosis, formatting scar tissue, as we can see a large number of white myocardial fibrous tissues in our experiment (supplementary data 1A). Second, hemodynamics will be one of the most crucial causes of the heart and renal injury. As cardiac output decreases, renal perfusion decreases, which leads to a decrease in ensuing glomerular filtration rate and increase in renal vascular resistance [23]. As a result, we established a model of ischemia and hypoxia in vitro. What is more, in our experiment, we found that the level of KIM1 and NGAL increased, which are all sensitive indicators of tubular injury [24, 25]. Therefore, in vivo and vitro, we detected the damage markers and autophagy level in proximal renal tubules. It may be that renal tubules are more sensitive to hypoxia than glomerulus; as a result, renal tubules are damaged before glomeruli [19]. Autophagy is another vital link in the study of pathological changes after myocardial infarction, playing a vital role to protect the kidney or heart from injury [26, 27]. However, few studies have observed autophagy changes in the heart and kidney at the same time. In our research, we found that the change of autophagy in the heart was consistent with the kidney. This is important for the use of autophagy in the treatment of cardiorenal syndrome because autophagy acts in one direction. Guanghua et al. [28] found that the autophagy activity reduced and lysosomal accumulation increased in the peripheral leukocytes of AMI patients. It appeared that autophagy may be a whole-body reaction. From this point of view, it is particularly important to study the mechanism of autophagy changes in order to better apply it to clinical treatment.

Our results suggested that the change of autophagy in the kidney was consistent with the heart, and autophagy was a process of dynamic change. From week 2 to week 4, LC3-II changed from a significant increase to a slight rise, and p62 changed from no obvious change to a significant increase. The accumulation of p62 may on account of autophagy induce decrease or lysosomal degradation dysfunction with the aggravation of disease. Subsequently, we detected autophagy-induced related proteins, ATG5, and Beclin1. At week 2, the expression of ATG5 and Beclin1 increased dramatically, while when it came to week 4, they increased lightly. As the activity of autophagy-induced increased, the degradation of p62 was increased at week 2. When it came to week 4, the autophagy-induced was insufficient, and p62 was increased. These showed that the autophagy induction was increased at week 2 and insufficient at week 4. While the changes of the renal function were that the uKIM1 in the model group was significantly higher at week 2, when it came to week 4, the uKIM1, uNGAL, and urinary albumin levels markedly increased (Figure 1(a)). The above results indicated that when the autophagy induction increased, it can protect the kidney from injury. These changes may be related to ischemia and hypoxia causing an adaptive increase in autophagy. As for experiments in vitro, the AC16 and HK-2 cells were incubated in a hypoxia incubator for 24 hours, and damage indicators increased significantly, while no significant difference occurred between autophagy-related indicators (data not listed). When we changed the incubation time to 4 hours, autophagy began to increase adaptively. These elucidated that autophagy was induced in the early period of hypoxia, and with the increase in time, the autophagy gradually weakened. What is more, when the cells were incubated with 3-MA, the autophagy inhibitor, the damage markers increased. The adaptive autophagy played a protective role in the kidney and heart after AMI, which may be involved in various mechanisms.

Autophagy degraded the damaged organelles caused by ischemia and hypoxia to protect myocardial and renal cells from injury and apoptosis. Scholars have found that the autophagy not only nonselectively degrades the metabolic waste to recycle nutrients and generate energy [29], but also highly selectively degrade misfolded and damaged proteins, including aggrephagy, mitophagy, lisophagy, and xenophagy. In particular, when the cells are under a hypoxic condition or continuous oxidative stress, or when a large amount of damaged mitochondria and other organelles are generated by ROS, autophagy can degrade the damaged organelles to maintain the homeostasis of the cells [30]. After the autophagosome contained the substance to be removed being mature, it combined with the lysosome to form an autolysosome, and then, under the action of the lysosomal hydrolase, the autophagosome and its contents are degraded. Interestingly, our previous research reported that autophagy was inactivated by lysosome dysfunction, which aggravated the injury of renal tubular epithelial cells [15, 31, 32] and podocytes [33]. Persistent ischemia and hypoxia may lead to lysosomal damage and autophagy inactivation, thereby accelerating cellular aging and initiating the cell death pathway. On the other hand, there are some common regulatory molecules between autophagy and apoptosis [34]. Studies have shown that under pathological conditions, BCL-2, a protein that inhibits apoptosis, can be dissociated from Beclin1, so that Beclin1 can induce autophagy and then inhibit apoptosis [35, 36]. However, the continuous cell stress causes the apoptotic signals caspase-3 and caspase-8 to be activated, which can cleave the N- and C-terminal of Beclin1 to make it unable to induce autophagy [37, 38]. This appears to be one of the reasons for the decrease of autophagy induction at week 4 after AMI compared with week 2.

In conclusion, this study provided experimental evidence for the early intervention of autophagy in AMI. While, to strength the conclusion, it would be worth to improve the animal experiment treated with 3-MA. It may better demonstrate the role of adaptive autophagy in rats with AMI. In the following experiments, we will carry out related research. Furthermore, underlying mechanisms of adaptive autophagy protecting the heart and kidney were still unclear, which worth more in-depth discussion in the future.

## Figures and Tables

**Figure 4:**
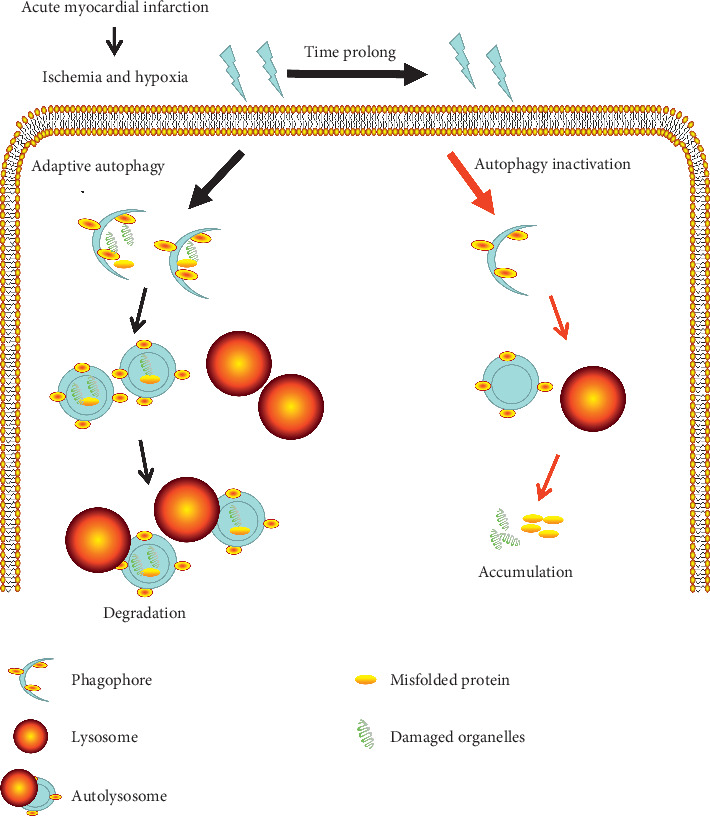
Schematic representation of cardiorenal autophagy response and effect in rats with myocardial infarction. This figure intuitively reflects that autophagy is a dynamic process. After AMI, the heart and kidney are in a state of ischemia and hypoxia. Initially, autophagy responds adaptively to degrade misfolded protein and damaged organelles to protect cells from damage. However, with time prolonging, persistent ischemia and hypoxia lead to autophagy inactivation, and then, the misfolded protein and damaged organelles could not be effectively degraded, thereby accelerating cellular aging and initiating the cell death pathway.

**Table 1:** Hemodynamic changes in the heart of rats after AMI.

	Week 2		Week 4	
	Sham	Model	Sham	Model
LVSP	149.2 ± 8.2	108.7 ± 9.6^*∗∗*^	170.4 ± 5.2	129.6 ± 4.5^*∗∗∗*^
LVEDP	−3.4 ± 4.3	5.5 ± 3.3	−9.5 ± 1.0	31.3 ± 5.5^*∗∗∗*##^
+dP/dt max	5495.0 ± 349.4	2865.2 ± 259.5^*∗∗∗*^	6209.5 ± 233.2	2418.0 ± 213.0^*∗∗∗*^
-dP/dt max	−4618.5 ± 331.8	−2526.3 ± 249.5^*∗∗*^	−5292.6 ± 247.8	−2405.4 ± 126.4^*∗∗∗*^

^*∗∗*^ *p* < 0.01; ^*∗∗∗*^*p* < 0.001, model compared with sham. ^##^*p* < 0.01, model of 2 weeks compared with 4 weeks. LVSP, left ventricular systolic pressure; LVEDP, left ventricular end-diastolic pressure; ±dp/dtmax, maximum rate of pressure rise/drop in the left ventricle. Data are expressed as the mean ± SEM (*n* = 6).

## Data Availability

The data used to support the findings of this study are available from the corresponding author upon request.

## Supplementary Materials

Supplementary Materials Supplementary 1: Morphology changes in the heart and kidney of rats after AMI. (A) The image of cardiac anatomy. Compared with the sham group, the left ventricular anterior wall and apex of the model group had obvious infarction, the infarct area became white, and the apex collapsed, particularly at week 4. Scale bar = 5 mm, n = 6. (B) Morphology in the heart and kidney. PAS staining, Masson's trichrome staining, and H&E staining of rats. Masson's staining showed that the area of fibrosis was greater in the model group at weeks 2 and 4 in the heart, but there was no significant fibrosis in the kidney. Scale bar: 50 *µ*m, n ≥ 5.
