# Supplementary material for: Adaptive Autophagy Offers Cardiorenal Protection in Rats with Acute Myocardial Infarction
Source: Cardiol Res Pract. 2020 Jun 20;2020:7158975. doi: 10.1155/2020/7158975 (PMC7322605; doi:10.1155/2020/7158975)
Supplement: Supplementary Materials — Supplementary 1: Morphology changes in the heart and kidney of rats after AMI. (A) The image of cardiac anatomy. Compared with the sham group, the left ventricular anterior wall and apex of the model group had obvious infarction, the infarct area became white, and the apex collapsed, particularly at week 4. Scale bar = 5 mm, n = 6. (B) Morphology in the heart and kidney. PAS staining, Masson's trichrome staining, and H&E staining of rats. Masson's staining showed that the area of fibrosis was greater in the model group at weeks 2 and 4 in the heart, but there was no significant fibrosis in the kidney. Scale bar: 50 µm, n ≥ 5. [file 7158975.f1.docx]

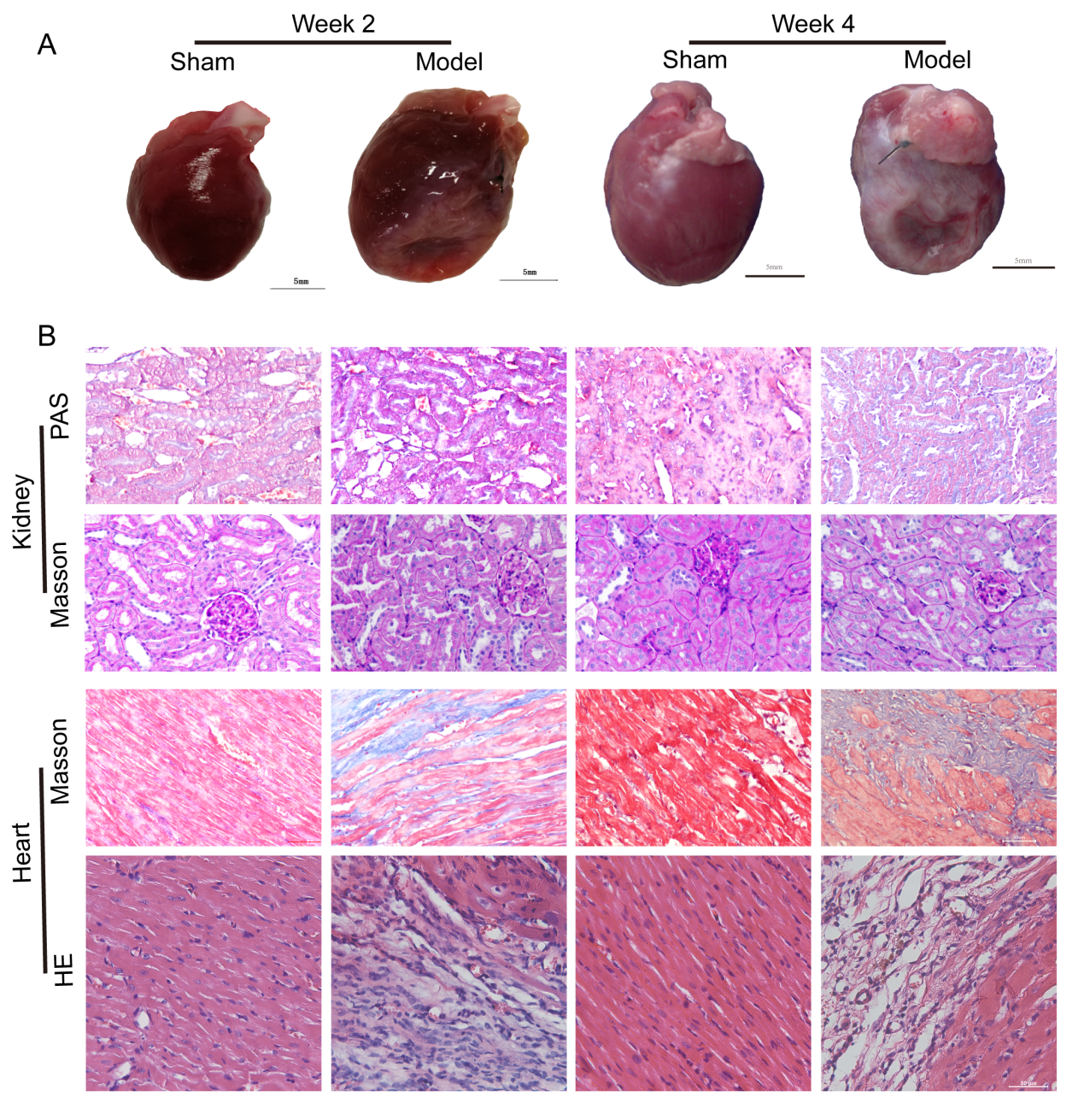


**Supplement 1.** Morphology changes in the heart and kidney of rats after AMI.

(A) The image of cardiac anatomy. Compared with the sham group, the left ventricular anterior wall and apex of the model group had obvious infarction, the infarct area became white, the apex collapsed, particularly at week 4. Scale bar = 5 mm, n = 6. (B) Morphology in the heart and kidney. PAS staining, Masson’s trichome staining and H&E staining of rats. Masson’s staining showed that the area of fibrosis was greater in model group at weeks 2 and 4 in the heart, but there was no significant fibrosis in the kidney. Scale bar: 50 µm, n ≥ 5.
